# Functions of Group 2 Innate Lymphoid Cells in Tumor Microenvironment

**DOI:** 10.3389/fimmu.2019.01615

**Published:** 2019-07-10

**Authors:** Jia Xiong, Haofei Wang, Jia He, Qingqing Wang

**Affiliations:** ^1^Institute of Immunology, Zhejiang University School of Medicine, Hangzhou, China; ^2^Department of Pharmacology, China Medical University School of Pharmacy, Shenyang, China

**Keywords:** group 2 innate lymphoid cells, tumor microenvironment, cytokines, immunomodulation, cancer immunotherapy

## Abstract

Innate lymphoid cells (ILCs), defined as a heterogeneous population of lymphocytes, have received much attention over recent years. They can be categorized into three subsets according to the expression profiles of transcription factors and differing levels of cytokine production. These cells are widely distributed in human organs and tissues, especially in mucosal tissue. The ILCs are involved in various physiological and pathological processes, including inflammation, worm expulsion, autoimmune disease and tumor progression, many of which have been investigated and clarified in recent studies. In the tumor microenvironment, group 2 innate lymphoid cells (ILC2s) have been proved to be able to either promote or inhibit tumor progression by producing different cytokines, recruiting diverse types of immune cells, expressing immunosuppressive molecules and by regulating the expression of certain inflammatory factors. This review summarizes recent research progress on the immunomodulatory functions of ILC2s in the tumor microenvironment and puts forward some perspectives for future study.

## Introduction

Innate lymphoid cells (ILCs) are heterogeneous and highly conserved immune cell populations that occur during organism development. These cells develop with negative lineage markers (Lin^−^). They lack phenotypical markers, antigen specificity and recombination-activating gene (Rag)-dependent rearrangement of antigen receptors (1, 2). A consensus has been reached in more recent studies that ILCs can be generally divided into non-cytotoxic ILCs and cytotoxic ILCs. Cytotoxic ILCs are named as conventional natural killer (NK) cells (3). Based on the differential requirements for transcriptional factors and cytokine production, the non-cytotoxic ILCs can be further categorized into three groups: group 1 innate lymphoid cells (ILC1s); group 2 innate lymphoid cells (ILC2s); and group 3 innate lymphoid cells (ILC3s) (4, 5). Main phenotypic markers of the different subsets of murine and human ILCs are well-summarized by Vivier et al. (6).

Non-cytotoxic ILCs exhibit similar functions to CD4^+^ helper T (Th) cells (7) including ILC1's constitutive expression of the transcriptional factor T-bet mirroring the function of Th1 cells. ILC1s can secret interferon-γ (IFN-γ) and tumor necrosis factor (TNF-α) when stimulated by interleukin 12 (IL-12). ILC2s consist of nuocytes, natural helper cells (NH cells) and innate helper 2 cells (IH2s). The development of ILC2s depends on the transcriptional factors GATA-3 and retinoic acid receptor-related orphan receptor-α (RORα), mirroring the nature of Th2 cells. Furthermore, these cells produce type 2 cytokines such as IL-4, IL-5, IL-9, IL-13, IL-15 in response to IL-33, IL-25, and thymic stromal lymphopoietin (TSLP). ILC3s, resembling Th17 cells, are comprised of lymphoid-tissue inducer cells (LTi cells), natural cytotoxicity receptors^−^ (NCR^−^s) ILC3s as well as NCR^+^ ILC3s. Collectively, ILC3s secrete cytokines such as IL-17 and IL-22. The RORγ is expressed in ILC3s when stimulated by IL-7 (4). The expanding family of ILC, like ILC22, ILC17 have been identified (8). Due to their plasticity (9), conversion of ILCs occur when they encounter distinct cytokines, transcription factors or distribute diversely. Human ILC1s show the potential capacity to convert into ILC3s in the presence of IL-23. IL-1β and retinoic acid have both been shown to accelerate that process (10). IL-25-responsive ILC2s populations in the lungs can differentiate into ILC3-like cells and produce IL-17 (11). On the contrary, the conversion of ILC3s to ILC1s can be induced by IL-2, IL-12, or IL-15 (12).

ILCs can be composed of cells of fetal, postnatal, or adult origins (13), the transcriptional factors they depend on are different (14). Adult ILCs are derived from the common lymphoid progenitors (CLPs) expressing CD127 in the bone marrow. CLPs give rise to early innate lymphoid progenitors (EILPs), which express integrin α4β7 under the participation of Notch signaling, transcription factors T cell factor 1 (TCF-1), thymocyte selection-associated HMG box protein (TOX) or nuclear factor interleukin-3 (Nfil3, also known as E4BP4). Through GATA-binding protein 3 (GATA3) and the inhibitor of DNA binding 2 (Id2) dependent pathways, EILPs develop into the common helper-like innate lymphoid progenitors (ChILPs), which are defined as lineage^−^IL-7R^+^Flt-3^−^integrin α4β7^+^CD25^−^Id2^high^ cells and have been shown to express a high level of Id2 and promyelocytic leukemia zinc finger (PLZF). ChILPs subsequently differentiate into lineage^−^IL-7R^+/−−^Flt-3^−^integrin α4β7^+^Id2^+^GATA-3^+^PLZF^+^ common innate lymphoid progenitors (CILPs), and then give rise to ILC precursors (ILCPs). NK progenitors are directly derived from EILPs and LTi progenitors not expressing PLZF come from ChILPs in a RORγt-dependent manner. In addition, LTi progenitors express a lower level of Id2, thymocyte selection-associated HMG box protein (TOX) and TCF-1, in contrast to CILPs. ILCPs generate different branches of the ILC family (15, 16). Human ILCs are more abundant in fetal tissues and cord blood than those in adults, while in ILC2s from bone marrow, compared with fetal and perinatal, development may differ considerably (17, 18). Also, fetal ILC2s are distributed to tissues before birth, postnatal ILC2s are generated in the period from birth through weaning, and adult-derived ILC2s slowly dilute the preexisting ILC2s (13). Fetal ILC precursors reside in the intestine during Peyer's patch development, and become a localized source of ILCs (17). ILC2s colonize tissues perinatally and are exceedingly long lived (19, 20), most of the ILC2s produced in bone marrow only are colonized in peripheral tissues in response to inflammatory signals (21–23).

ILC2s have received much attention in areas of allergies, worm expulsion, tissue repair and adipose metabolism homeostasis (4, 24). In recent years, increasing studies have focused on the roles of ILC2s in regulating tumor growth and cancer's inflammatory microenvironment. Here, we summarize the research progress and new findings in clarifying the functions and significance of ILC2s in the tumor microenvironment (TME).

## The Physiological Characters of ILC2s

ILC2s are mainly distributed in the mucosal tissues of the skin, lungs and the gastrointestinal tract, with a small amount of ILC2s also existing in peripheral blood. ILC2s are involved in the regulation of many physiological and pathological processes including asthma, allergies, atopic dermatitis, worm expulsion, tissue repair, adipose tissue regulation, metabolic homeostasis, and tumor growth (4). Moreover, ILC2s can be further classified into natural ILC2 cells (nILC2) (which are sensitive to IL-33), and inflammatory ILC2 cells (iILC2) (which are sensitive to IL-25) (11). Immunological memory has long been described in the adaptive immune system, and now has been related to ILC2s and termed “trained immunity.” In this, naïve ILC2s become memory ILC2s upon their encounter with alarmins, where they remember previous activations and respond more vigorously upon subsequent reactivations (25, 26).

The principal transcriptional factors, implicated in the differentiation and development of ILC2s, are GATA3 and RORα, which share the similar function acting in parallel (27). Other transcription factors also take part in the regulation of the development of ILCs, including B-cell CLL/lymphoma 11b (Bcl-11b) (28–30), TCF-1 (15), growth factor independent 1 transcription repressor (GFI1) (31) and E-twenty six 1 (ETS-1) (32).

ILC2s express surface molecules, including MHC II (33), inducible co-stimulatory molecule ligand/inducible co-stimulatory molecule (ICOSL/ICOS) (34), programmed death-ligand 1 (PD-L1) (35), CRTH2 (36), NKp30 (37), killer cell lectin-like receptor subfamily G member 1 (KLRG1) (11) and OX40 (38). In response to IL-25, IL-33, and TSLP, which are mostly derived from epithelial cells, ILC2s are activated to produce the type 2 cytokines IL-4, IL-5, IL-9, IL-13, and other factors such as methionine-enkephalin (Met-Enk) and Amphiregulin (Areg), which suggests that ILC2s exhibit similar functions as CD4^+^ Th2 cells (5). Some studies have reported that ILC2s role in antigen presentation is having a dialog with CD4^+^T cells via the expression of MHC II on the surface of ILC2s (39).

Moreover, recent studies have described many new molecules which play important roles in promoting the maturation and development of ILC2s. E3 ubiquitin ligase Von Hippel-Lindau (VHL)-deficiency in innate lymphoid progenitors cause a decrease of mature ILC2s in peripheral non-lymphoid tissues, and VHL-deficient ILC2s exhibit enhanced expression of hypoxia-inducible factor 1α (HIF-1α) and subsequently caused enhanced glycolysis. VHL-deficiency results in low expressions of the IL-33 receptor ST2 and attenuated IL-33-induced ILC2 development has also been described. HIF-1α drives the expression of the glycolytic enzyme pyruvate kinase M2, which downregulates the expression of ST2 via reduced H3K4me3 modification at the *Gata3* promoter and *Il5* gene loci in VHL-deficient ILC2s (40). The Intercellular adhesion molecule-1 (ICAM-1) is required for the development of ILC2s, where ICAM-1^−/−^ ILC2s show impaired ERK signaling which leads to the diminished expression of GATA3 and production of type 2 cytokines (41). In the gastrointestinal tract, murine ILC2s were demonstrated to be co-localized with cholinergic neurons expressing the neuropeptide neuromedin U (NMU) that could be recognized by the receptor NMUR1 on ILC2s. NMUR1 is important for the stimulation of the secretion of the type 2 cytokines IL-5, IL-9, and IL-13 in ILC2s (42–44). In addition, the aryl hydrocarbon receptor (Ahr) is expressed in gut ILC2s and has been implicated in controlling chromatin accessibility at the Ahr loci, playing a role in restricting the production of IL-5, IL-13, and amphiregulin via intestinal ILC2s in a cell-intrinsic manner. Ahr inhibits the expression of the IL-33 receptor ST2 in ILC2s, thereby suppressing the transcription factor—growth factor independent 1 transcription repressor (GFI1) (45). Type I interferon was reported to restrict type 2 immunopathology by diminishing type 2 signature cytokine production, cell proliferation and increasing cell death of ILC2s dependent on the interferon stimulated gene factor 3 (ISGF3) (46).

## The Functions of ILC2s in the Tumor Microenvironment

### ILC2s-Derived Cytokines Regulate the Growth of Tumors

As shown above, ILC2s can secrete cytokines IL-4, IL-5, IL-9, and IL-13. When ILC2s respond to IL-33, the release of high levels of IL-13 can lead to the occurrence of cholangiocyte hyperplasia and epithelial repair. In the bile duct of mice, the induction of the IL-33/ILC2/IL-13 pathway by activated AKT and Yes-associated protein (YAP) further induces cholangiocarcinoma (47). In addition, IL-13 from ILC2s plays a critical role in recruiting myeloid-derived suppressor cells (MDSCs) and M2 phenotype macrophages, which establish an immunosuppressive microenvironment to restrain anti-tumor responses (48). Dendritic Cells (DCs) are potent professional antigen-presentation cells, linking innate immunity with adaptive immunity by presenting tumor antigens to T cells to elicit a specific anti-tumor immunity. IL-13 production by ILC2s is critical for the migration of CD40^+^ activated DCs from the lung to drainage in the lymph nodes inducing Th2 immunity (49, 50).

During tumor development, the IL-4 receptor α (IL-4α) is overexpressed in many epithelial cancers. Recent studies have found that the production of IL-4 by ILC2s enhances the proliferation and metastasis of epithelial cancer cells (51–55). Other cytokines derived from ILC2s such as IL-5, have also been described to elicit an anti-tumor effect by recruiting eosinophils, which further attract CD4^+^ T cells and CD8^+^ T cells by producing chemokine (C-X-C motif) ligand 9 (CXCL9), CXCL10, chemokine (C-C motif) ligand 5 (CCL5) (via STAT1), CCL17 and CCL22 (via STAT6) (48, 56).

The role of IL-9 in tumors is controversial. Deficiency or neutralization of IL-9 appears to promote melanoma growth (57, 58). In contrast, overexpression of IL-9 is related to poor prognosis in some hematopoietic carcinomas (59). Recent study suggests that IL-9, as released by ILC2s, can induce specific cytotoxic T lymphocyte (CTL) responses by enhancing the cross-presentation of DCs and reduce tumor metastasis (60).

Epidermal growth factor receptor (EGFR) antagonists are used in treating epithelial-derived cancers. Interestingly, the ILC2-derived EGFR ligand, Areg, was found to support the growth of lung cancer cells and to inhibit cell apoptosis (12, 61). In the peripheral blood of gastric cancer patients, the increased mRNA expression levels of arginase 1 (ARG1) and inducible nitric oxide synthase (iNOS), which represent immunosuppressive factors, are also correlated with the increased expression of the ILC2-related markers RORα, T1/ST2, IL-17RB, CRTH2, ICOS, CD45, and the signature cytokines IL-13 and IL-5 (62). ILC2-derived Arg1 was also reported to inhibit T cell responses (63). The role of IL-33 in promoting hypoxic TME by inducing aberrant angiogenesis has also been recently highlighted (64). Subsequent to this, the high production of reactive oxygen species (ROS) elicits the expression of chemokine (C-X-C motif) receptor 2 (CXCR2) on tumor cells. Notably, ILC2s-derived CXCL2 has been shown to induce the strong apoptosis of EL4 lymphoma cells via CXCR2 (64).

### ILC2s-Mediated Recruitment of Immunosuppressive Cell Subsets

The number of ILC2s increases in TME, and increased IL-13 derived from ILC2s leads to the accumulation of monocytic myeloid-derived suppressor cells (M-MDSCs) to support cancer progression (36, 65). MDSCs can promote tumor growth by expressing multiple pro-angiogenesis characteristics, and they suppress T cells and NK cells by expressing a high level of Arg1, iNOS, and reactive Oxygen species (ROS). IL-13 derived from ILC2s helps to activate Monocytic-MDSCs (M-MDSCs) after stimulation by IL-33 [8, 33], whilst, IL-33 itself can directly elicit the expansion of M-MDSC (66–68). In addition, ILC2s can also induce MDSCs to produce transforming growth factor-β (TGF-β), which contributes to the alternative activation of macrophages (69).

Regulatory T cells (Tregs) manifest their suppressive characteristics by secreting immunosuppressive factors such as IL-10, IL-35, and TGF-β (70), inducing the expression of inhibitory receptors like cytotoxic T lymphocyte antigen 4 (CTLA-4) (70), killing effector cells via granzyme B (71, 72) and perforin (73), interfering with the cellular metabolism, and down-modulating expression of CD80 and CD86 on DCs to support tumor metastasis and evasion from the immune system (74). Previous studies have shown that ILC2s activate Tregs to establish and maintain an immunosuppressive microenvironment through producing Areg, the ligand of epidermal growth factor receptor (EGFR) on Tregs (12, 48, 75). Importantly, ILC2s promote the accumulation of Tregs via the interaction of ICOSL and ICOS, which are both expressed on the surface of ILC2s (76). Nonetheless, Tregs conversely inhibits the function of ILC2s through restraining the binding of ICOS/ICOSL (34, 77).

Macrophages have critical functions in the stroma milieu of tumors. They show clear heterogeneity with different phenotypes appearing upon exposure to different stimuli. Generally, macrophages can be classified into M1 and M2 subsets. Infiltration of M2-like tumor-associated macrophages (TAMs) promotes tumor progression via the suppression of anti-tumor immune responses. Activated ILC2s produce IL-13 which play a key role in the induction of the polarization of the M2 phenotype via the STAT6 signaling pathways, and which contribute to the survival of tumors (12, 66, 77, 78).

### Programmed Death-1 (PD-1)/PD-L1 Signaling Restrains the Activation of ILC2s

PD-1 is an inhibitory receptor expressed on the surface of T cells, it is also expressed on the surface of ILC2s (79). The increased PD-1 expression on ILC2s correlates with responses to IL-33. The combination of PD-1 and PD-L1 provides a negative signal which limits the proliferation and cytokine secretion of mature ILC2s by inhibiting the phosphorylation of STAT5. In this way, the immunoregulation functions mediated by mature ILC2s are impaired (35, 80, 81). However, far too little attention has been paid to ILC2-mediated immunomodulation in tumors by PD-1 and PD-L1. More detailed studies are required to investigate such relationships.

### Prostaglandin Regulates the Functions of ILC2s

The prostaglandin (PG) D2 receptor, chemoattractant receptor-homologous molecule (CRTH2), is expressed on Th2 lymphocytes and also exists on the surface of human ILC2s (7). The activation and migration of ILC2s, together with the production of type 2 cytokines by ILC2s, can be ascribed to the binding of PGD2 to CRTH2 (24). In addition, ST2 (IL-33 receptor) and IL-17RA (IL-25 receptor) are also expressed on ILC2s (82). Tumor-derived PGD2 and the natural cytotoxicity receptor 3 (NCR3) ligand B7H6, respectively react with CRTH2 and NCR NKp30 expressed on ILC2s (83), inducing ILC2s, to activate M-MDSC by secreting IL-13 which exhibits the immunosuppressive functions mentioned above (4, 37). As for patients with acute promyelocytic leukemia (APL), specifically impairing the immunosuppressive axis PGD2/IL-13/NKp30 can partially decrease the numbers of ILC2s and M-MDSC to ameliorate survival (36). However, PGE2 reduces the proliferation of ILC2s by interacting with the receptors EP2 and EP4 expressed on ILC2s, and so the expression of GATA3 and the secretion of type 2 cytokines can also be restrained (84). As discussed above, different prostaglandins act uniquely on ILC2s to render distinct effects on tumor progression. Thus, prostaglandins have emerged as the potential targets of cancer therapy. However, the function of prostaglandins to control the regulating effect of ILC2s on tumors still needs further in-depth investigation.

## Perspectives

**Figure 1:**
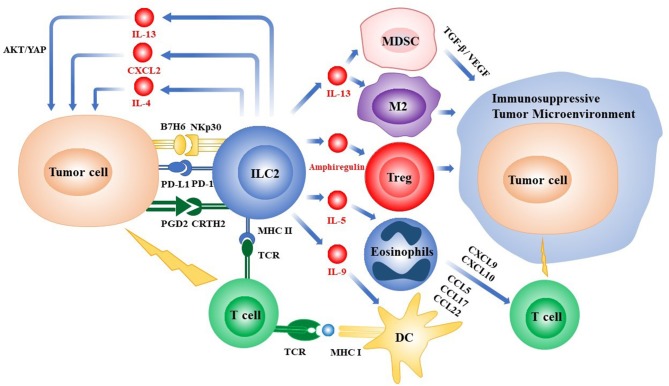
Overview of the roles of ILC2s in the tumor microenvironment. ILC2s can directly regulate the growth and metastasis of tumor cells by secreting cytokines or via the interactions of receptors and ligands expressed on the various types of cells, respectively. ILC2s can also indirectly influence the fates of tumors by recruiting immune cells into the tumor microenvironment or promoting the process of tumor-antigen presentation. ILC2s, group 2 innate lymphoid cells; PD-1/PD-L1, programmed death-1/programmed death-ligand 1; Treg, regulatory T cell; IL, interleukin; MHC, major histocompatibility complex; CXCL, chemokine (C-X-C motif) ligand; CCL, chemokine (C-C motif) ligand; TCR, T cell receptor; DC, dendritic cell; YAP, Yes-associated protein; MDSC, myeloid-derived suppressor cell; M2, M2-like macrophage; TGF-β, transforming growth factor-β; VEGF, vascular endothelial growth factor; PGD2, prostaglandin D2; CRTH2, chemoattractant receptor homologous to the T helper 2 Cells.

In recent years, ILC2s have attracted much attention. They are important players in the modulation of the tumor microenvironment and regulate the host's antitumor immunity (Figure 1). Of note, tumor-associated ILC2 phenotypes have been reported in various types of cancer. However, a better understanding of the interaction between these ILC2s and the TME is required (3). The expression of surface markers on ILC2s is diverse in the context of the distinct TME, indicating a high heterogeneity in ILC2s. It is essential to identify the reliable and specific cell surface markers and to understand how the TME influences the phenotypic heterogeneity and functional diversity of ILC2s. Furthermore, some issues related to the interaction of ILCs with the TME remain to be addressed including those of distinct origins and periods of tumor progression, metabolic cross-talks between tumor and ILC2s, and the interaction between ILC2s and other immune cells in the TME, especially CD8^+^ T cells.

Understanding the phenotypic plasticity of ILC2s will be of benefit toward answering many questions relating to conversions among the three groups of the ILC family. When does the ILC conversion occur? Do epigenetic modification mechanisms exist during the process? Will the conversion be clinically meaningful if tumor-promoting ILC2s can adopt an anti-tumorigenic phenotype? Overall, little is known regarding how epigenetic modifications control the unique gene expression patterns and biological functions of ILC2s in the TME. Whether tumor-associated ILC2-derived cytokines or exosomes lead to the formation of the TME or pre-metastatic niche also still remains ambiguous. Future work in the field is required to address these key issues.

Given the preferential distribution of ILC2s in mucosal tissue, and the important role the microbiota plays in homeostasis of immunity, further research relating to crosstalk between ILC2s and the microbiota that reside in the microenvironment of lung cancer, colorectal cancer and gastric cancer may also pave the way for a new strategies of cancer immunotherapy (85).

With the deepening of research, ILC2s may have the potential to be a feasible immunotherapeutic target for optimizing the tumor immunotherapy strategies. This highlights the need to further identify their critical features in the inflammatory tumor microenvironment.

## Author Contributions

JX, HW, and JH have participated in editing the article. QW provided intellectual guidance. All the authors have read the final version of the paper and approved it for publication.

### Conflict of Interest Statement

The authors declare that the research was conducted in the absence of any commercial or financial relationships that could be construed as a potential conflict of interest.
